# Space Charge-Limited Current Transport Mechanism in Crossbar Junction Embedding Molecular Spin Crossovers

**DOI:** 10.1021/acsami.0c07445

**Published:** 2020-06-18

**Authors:** Giuseppe Cucinotta, Lorenzo Poggini, Niccolò Giaconi, Alberto Cini, Mathieu Gonidec, Matteo Atzori, Enrico Berretti, Alessandro Lavacchi, Maria Fittipaldi, Aleksandr I. Chumakov, Rudolf Rüffer, Patrick Rosa, Matteo Mannini

**Affiliations:** † Department of Chemistry “U. Schiff” and INSTM Research Unit, University of Florence, Via della Lastruccia 3-13, Sesto Fiorentino, FI 50019, Italy; ‡ CNRS, University of Bordeaux, ICMCB, UMR 5026, Pessac 33600, France; ∥ Department of Physics and Astronomy and INSTM Research Unit, University of Florence, Via Sansone 1, Sesto Fiorentino, FI 50019, Italy; ⊥ Institute for Chemistry of OrganoMetallic Compounds (ICCOM-CNR), Via Madonna del Piano, Sesto Fiorentino, FI 50019, Italy; # ESRF-The European Synchrotron, Avenue des Martyrs 71, Grenoble 38000, France

**Keywords:** spin crossover, transport measurements, hybrid device, FIB-STEM, synchrotron Mössbauer spectroscopy, molecular magnetism, molecular spintronics

## Abstract

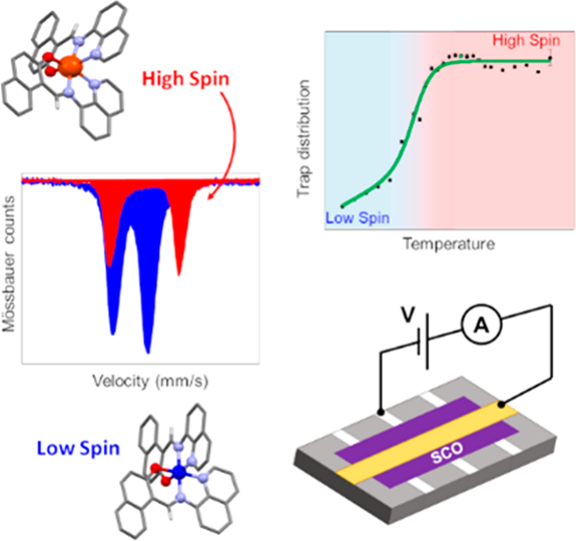

Spin crossover complexes are among the most studied classes of molecular switches and have attracted considerable attention for their potential technological use as active units in multifunctional devices. A fundamental step toward their practical implementation is the integration in macroscopic devices adopting hybrid vertical architectures. First, the physical properties of technological interest shown by these materials in the bulk phase have to be retained once they are deposited on a solid surface. Herein, we describe the study of a hybrid molecular inorganic junction embedding the spin crossover complex [Fe(qnal)_2_] (qnal = quinoline-naphthaldehyde) as an active switchable thin film sandwiched within energy-optimized metallic electrodes. In these junctions, developed and characterized with the support of state of the art techniques including synchrotron Mössbauer source (SMS) spectroscopy and focused-ion beam scanning transmission electron microscopy, we observed that the spin state conversion of the Fe(II)-based spin crossover film is associated with a transition from a space charge-limited current (SCLC) transport mechanism with shallow traps to a SCLC mechanism characterized by the presence of an exponential distribution of traps concomitant with the spin transition temperature.

## Introduction

Molecule-based materials represent a good alternative to conventional inorganic semiconductor materials for the development of innovative devices as a consequence of their rich tunability of the molecular properties.^1−3^ Moreover, the processability and the low weight of organic materials permit the production of flexible devices,^4,5^ a key aspect for next-generation devices. Spin crossover (SCO) complexes are molecular compounds able to reversibly switch their physical properties upon application of external stimuli (temperature, light irradiation, applied magnetic and electric fields, pressure, etc.). This is associated with a switch of the spin state between two magnetic states (low spin, LS, and high spin, HS) of a coordinated metal ion. These inorganic complexes have been proposed as active materials in functional devices with reversible magnetic and electrical response.^6−14^ To the best of our knowledge, only a few reported studies deal with the integration of SCO materials in electrical and electromechanical devices,^6,15^ since the preparation of high-quality thin films and electronically optimized hybrid architectures is critical. Most studies have been focused on rather thick films of highly insulating compounds that may operate in the hopping regime in order to limit problems associated with the device preparation, such us percolation and short-circuits issues.^10^ However, entering in the domain of spintronics, interfacial phenomena play a crucial role. In this context, it is important to increase the general knowledge on devices based on thinner molecular layers.^16^ Some of us have previously studied^12,13^ the transport properties of vertical junctions incorporating very thin films of SCO complexes formulated as [Fe(HB(trz)_3_)_2_] (HB(trz)_3_ = tris(1*H*-1,2,4-triazol-1-yl)borohydride) and [Fe(H_2_B(pz)_2_)_2_(phen)] (H_2_B(pz)_2_ = bis(pyrazol-1-yl)borohydride, phen = 1,10-phenanthroline), demonstrating that different transport mechanisms can occur. This has been done by use of a vertical junction architecture,^12,13^ based on a soft eutectic GaIn liquid top electrode (EGaIn),^17^ allowing a gentle contact with ultrathin films. In this context, we also recently showed that the ferrous complex [Fe(qnal)_2_] (Hqnal = quinoline-naphthaldehyde) is an ideal target toward integration of molecular spin crossover in vertical devices, since it can be deposited as a very smooth high-quality ultrathin film by thermal sublimation while maintaining its bulk properties with a slight shift (ca. 10 K) in the spin crossover temperature *T*_1/2_ observed ca. 220 K.^18^

In this paper, we report the development of an Ag//[Fe(qnal)_2_]//LiF//Au multilayer device to study the mechanism which determines the current flowing across these junctions as a function of temperature, with the support of gas-phase DFT calculations, electrochemistry, magnetization measurements, synchrotron Mössbauer source (SMS) spectroscopy, and focused ion beam scanning transmission electron microscopy (FIB-STEM). LiF was used as an intercalation layer between the molecular film and the top electrode to prevent unwanted short circuits across the molecular layer and to modulate the work function of the metallic electrodes.^19,20^ We show that in this device there is a change in the temperature dependence of the current density at a temperature at which the switching between HS and LS states occurs. More precisely, we show that the system is in a low-conductance state with no evident temperature dependence when the compound is in the LS state. When it switches to the HS state, the device then switches to a high-conductance state characterized by the presence of thermal activation mechanisms at higher temperature. In addition, analysis of the current density dependence on applied voltage reveals that the electric transport is described by a transition from space charge-limited current (SCLC) with shallow traps^21^ at low temperature to SCLC with an exponential trap distribution^21^ at high temperature.

## Results and Discussion

**Figure 1:**
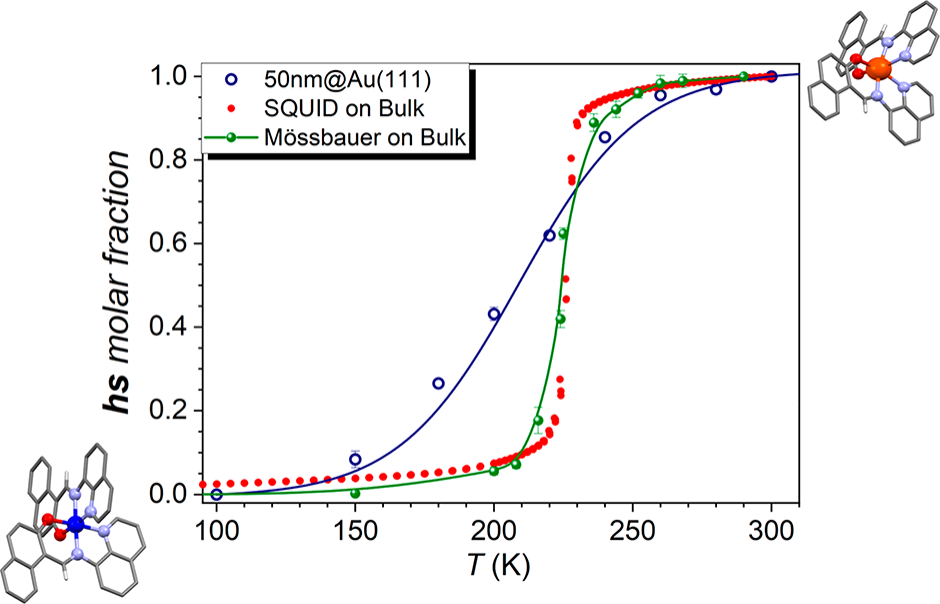
Comparison between bulk SQUID magnetic characterization (red dots), Mössbauer spectroscopy of [^57^Fe(qnal)_2_] in bulk (green dots, the line is a guide for the eyes), and high-spin Fe(II) thermal distribution profile obtained from XAS on a thin film (blue dots). Blue line is the best fit to a Boltzmann distribution of the XAS data giving *T*_1/2_ = 210 ± 5 K (originally reported in ref (18)); total electron yield mode used in ref (18) to characterize the LS–HS conversion is surface sensitive, thus providing information only on the topmost molecular layers and cannot be considered informative as the SMS spectroscopy respective to the entire deposit.

[Fe(qnal)_2_] was synthesized following the literature procedures,^22,23^ and the details are reported in the Supporting Information and in the Methods section (see Synthesis Section, Figures S1 and S2). [Fe(qnal)_2_] shows a molecular structure in which a hexacoordinated Fe(II) ion features a slightly distorted octahedral coordination geometry (insert in Figure 1). Thanks to four nitrogen atoms and two oxygen donor atoms, the qnal ligand field provides an appropriate strength to stabilize the diamagnetic LS state at low temperature (below 150 K) and the paramagnetic HS state at high temperature (above 250 K).^18,24^ The low-temperature phase is characterized by an Fe^II^ ion featuring an octahedral coordination geometry which is only slightly distorted by the coordination with the ligand with average M–N(O) distances of ca. 1.94 Å, while in the high-temperature phase, the coordination octahedron is highly distorted and associated with longer M–N(O) distances, on average ca. 2.11 Å. This is associated with the partial promotion of d electrons located in bonding/nonbonding orbitals (*t*_*2g*_) to those having an antibonding character (*e*_*g*_) when passing from the low- to the high-temperature phase.^25^ Consequently, the total volume of the unit cell reversibly changes from 1568 (low temperature) to 1619 Å^3^ (high temperature) without loss of crystallinity. Bulk magnetic measurements of [Fe(qnal)_2_] (see Figure 1) show an abrupt SCO behavior with a *T*_1/2_ (225 ± 1 K)^18^ close to that reported in the seminal work of Kuroda-Sowa and co-workers (218 K).^23^ Magnetic measurements on a microcrystalline powder of the same complex isotopically enriched in ^57^Fe, [^57^Fe(qnal)_2_] show an abrupt transition at 225 ± 1 K (see Figure S2). This thermal behavior has been confirmed by analysis of standard transmission Mössbauer spectra of bulk [^57^Fe(qnal)_2_], as reported in Figure 1 (green dots), pointing out a *T*_1/2_ = 225 ± 4 K. The complete series of the Mössbauer spectra as a function of temperature is reported in Figure S3.

Earlier reports evidenced that [Fe(qnal)_2_] has the possibility to induce the SCO through light irradiation (light-induced excited spin state trapping, LIESST, λ = 531 nm) at cryogenic temperature in bulk^23^ as well as once deposited as a thin film.^18^ It was also shown that after a thermal treatment at ca. 460 K at ambient pressure and under high-vacuum condition, release of the crystallization CH_2_Cl_2_ molecule leads to formation of the unsolvated form [Fe(qnal)_2_], which is accompanied by a modification of the *T*_1/2_.^18,23^

**Figure 2:**
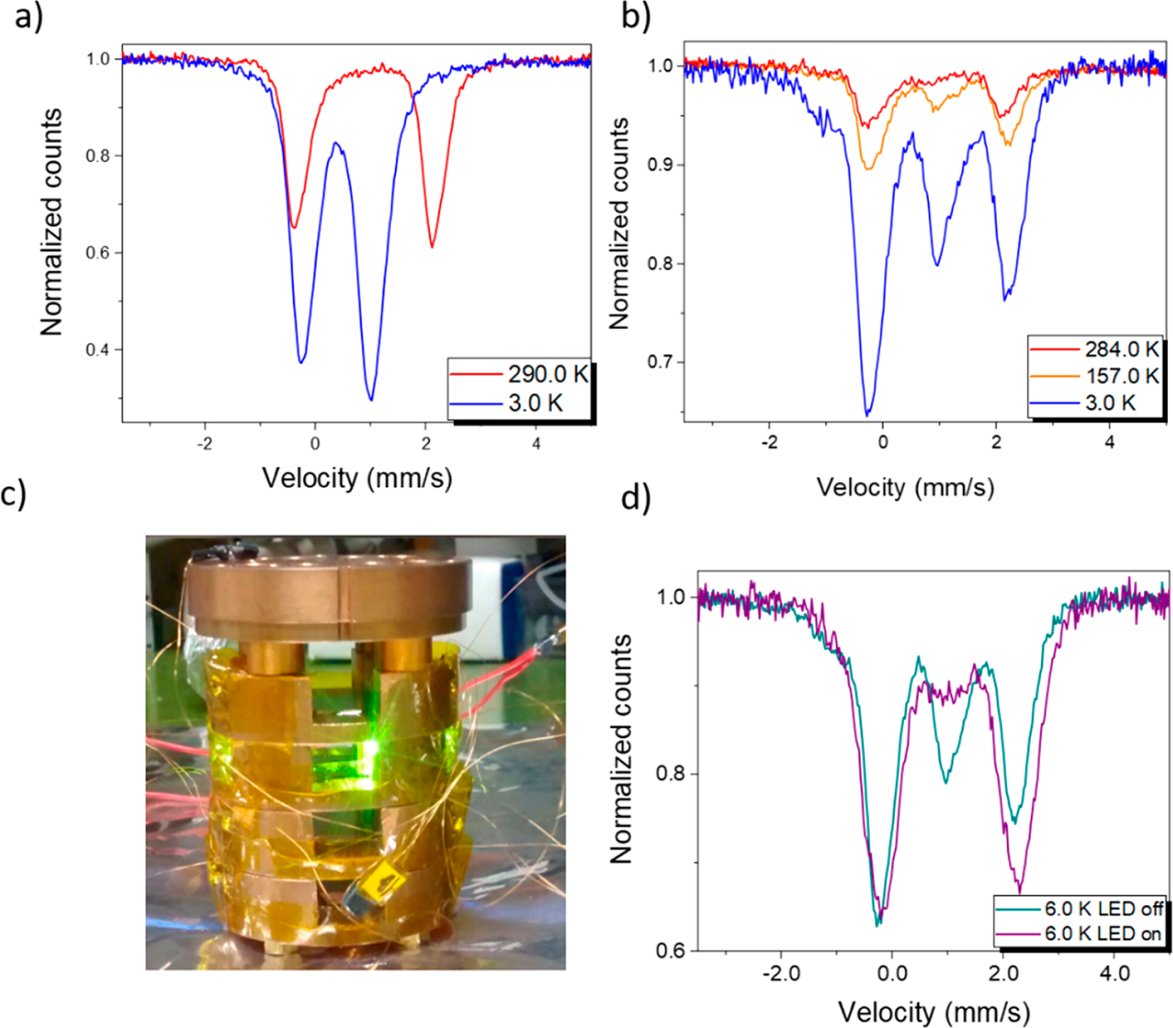
(a) Synchrotron Mössbauer source spectra of the drop cast sample of [^57^Fe(qnal)_2_] at 3.0 (blue) and 290 K (red). (b) Synchrotron Mössbauer source spectra of the 100 nm thick sublimated sample of ^57^[Fe(qnal)_2_] at different temperatures. Complete temperature trend is reported in Figure S4. (c) Image of the sample holder with LEDs mounted to obtain the LIESST measurements. (d) Synchrotron Mössbauer source spectra of the 100 nm thick sublimated sample of [^57^Fe(qnal)_2_] acquired at 6.0 K before and after irradiation with λ = 531 nm

A SMS spectroscopy study was performed initially on a thick film obtained by drop casting a concentrated solution of [^**57**^Fe(qnal)_2_] in CH_2_Cl_2_ and subsequently on a sublimated film. SMS spectra of the [^57^Fe(qnal)_2_] drop cast sample, whose average thickness, evaluated with the SMS fitting, was 360 ± 20 nm, are reported in Figure 2a, evidencing that a complete spin conversion is encountered, as in the bulk (Figure S3). At 290 K, a HS state characterized by an isomer shift δ = 0.890 ± 0.003 mm/s and a quadrupole splitting Δ*E*_Q_ = 2.496 ± 0.006 mm/s is present, while at 3.0 K a LS state with δ = 0.381 ± 0.001 mm/s and Δ*E*_Q_ = 1.198 ± 0.001 mm/s is found. The hyperfine values of the HS and LS states are comparable with those found using standard Mössbauer spectroscopy for [^57^Fe(qnal)_2_] in the bulk phase (see Table S1).

Moving to the sublimated 100 nm thick film (Figure 2b), it can be noticed that at 3.0 K there is still a high contribution of HS, as exemplified by the persistence in the spectrum of a third line above 2 mm/s. Similar hyperfine values are found for the HS and LS states, although characterized by different Gaussian distributions (see Table S1). Moreover, as one can notice from the fitting of the temperature trend (see Figure S4), the SCO efficiency is on the order of 20%. A reduction of the SCO efficiency when molecules are deposited on a substrate is not unexpected and can be associated with the interaction with the substrate as well as to a different organization of the sublimated molecules with respect to the bulk phase.^26−31^ A similar efficiency is encountered after LIESST with λ = 531 nm (for 10 min of irradiation with a total power 4 mW, Figure 2c and 2d). After light irradiation at 6.0 K the system reaches a HS fraction of 90 ± 6% (starting from an HS fraction of 70 ± 4% before irradiation), suggesting that the same fraction of molecules that can be converted with temperature can be also switched optically at low temperature. We previously observed a similar behavior on another SCO compound on the same substrate.^32^

**Figure 3:**
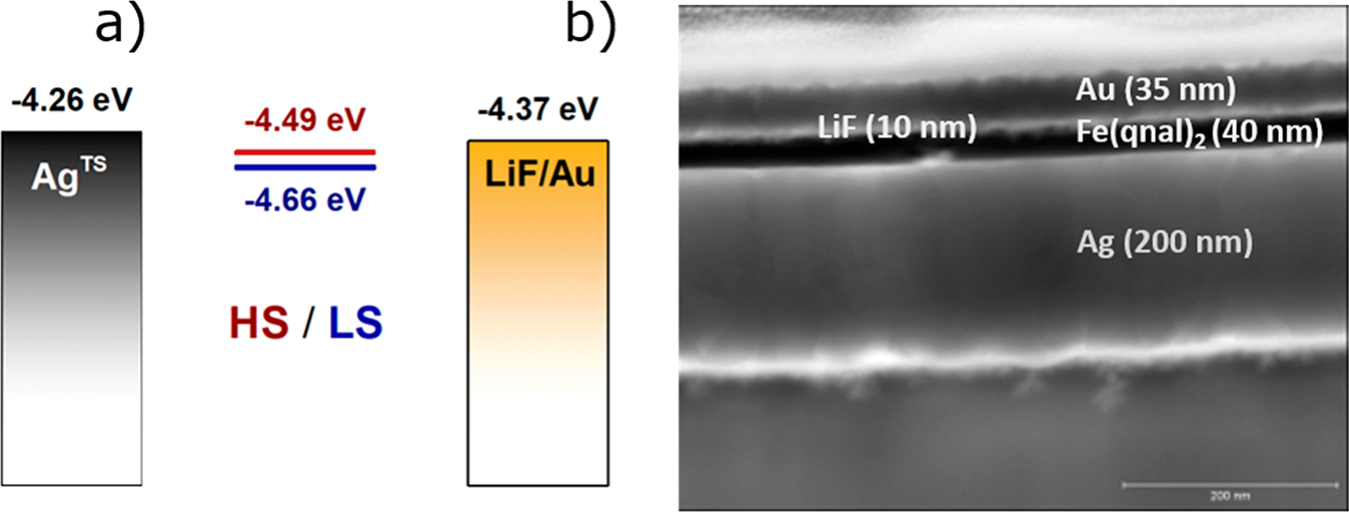
(a) Electronic configuration of the junctions in open circuit for the Ag and LiF/Au electrodes and for both HS (red line) and LS (blue line) states. (b) Dark-field STEM image of a thin section of a device with different layers and thicknesses indicated.

In order to have a complete picture of the expected functionality of [Fe(qnal)_2_] in a device and before assembling a vertical architecture embedding it, we performed gas-phase DFT calculations (see Methods for details) for [Fe(qnal)_2_] in both spin states and estimated the HOMO and LUMO orbital energies using Koopman’s theorem.^33^ As mentioned earlier, charge transport through thin dielectric films, in general, can occur via a variety of mechanisms. It follows, as we have shown recently, that varying the nature of the SCO compounds in vertical devices can lead to significant changes in the temperature-dependent characteristics, as some compounds present clear tunnel-like behaviors while others exhibit thermally activated transport. In order to properly describe the occurring processes, it is important to get reasonable estimates of the energy of the molecular orbitals involved in charge transport (in both HS and LS states). Our DFT analysis, as expected for Fe(II) complexes,^34^ evidences that the HOMO orbitals of both states (−4.66 and −4.49 eV for the LS and the HS states, respectively) are much closer in energy to the tabulated work functions of the Ag (−4.26 eV) and LiFAu (−4.37 eV) electrodes^19,35^ (see Figure 3a) than the respective LUMO orbitals (−3.24 and −3.19 eV, respectively). This points out that charge transport in [Fe(qnal)_2_] is likely to be dominated by hole transport. It is noteworthy that the estimated HOMO energies for [Fe(qnal)_2_]_**LS**_ and [Fe(qnal)_2_]_**HS**_ are quite high values when compared with other Fe(II) compounds^12,13^ and suggest that [Fe(qnal)_2_] could better act as a conducting layer than other systems previously used to assemble hybrid devices.^12,13^ To ascertain the accuracy of energy estimations, we performed in parallel room-temperature solution electrochemistry experiments (see Figure S5) to get an independent estimation of the HOMO orbital energy of the HS state (which should be populated almost quantitatively at room temperature). The Fe(II)/Fe(III) redox couple was therefore measured to be *E*_1/2_ = −0.62 vs Fc/Fc^+^ in dichloromethane containing 0.1 M *n*Bu_4_PF_6_ as supporting electrolyte. Assuming that *E*_HOMO_ ≈ *E*_NHE_ + *E*_1/2vsNHE_, we extracted a value of −4.52 eV for the HOMO of [Fe(qnal)_2_]_**HS**_, which is in excellent agreement with the values calculated by DFT.

As bottom electrodes for the vertical device, we used Ag template-stripped patterned layers which provide high-quality metal films with a very low roughness.^36^ This was crucial to limit short-circuit problems that are also often associated with the roughness of the metal films and the inhomogeneities in the molecular film. However, in this case, the latter has been excluded because of the high-quality ultrathin films of [Fe(qnal)_2_], as estimated by AFM analysis (Figure S6) of the deposited material. This analysis evidenced a nice defect-free SCO deposit excluding the presence of pin holes on the surface and Vollmer–Weber growth (figure S6c) and an RMS roughness of about. 1.1 ± 0.1 nm over an area of 23 μm^2^. The thickness of the “active” layer of [Fe(qnal)_2_] deposited on top of the Ag electrodes was about 50 nm (effective thickness equal to 47 nm estimated by an AFM test, see Methods). The stacked devices were completed, as described in the Methods section and reported in Figure S7, with the deposition of a ca. 35 nm thick Au crossbar. To avoid gold percolation through the molecular film that could lead to short circuits in the final device, a 10 nm LiF intercalation layer was evaporated before gold deposition.

One of the devices used for the transport measurements (see below) was a posteriori characterized morphologically by STEM imaging on a lamella, extracted from a device surface using focused ion beam methods. The nature of this procedure enabled the stratigraphic study of this ultrathin stack of heterogeneous materials, allowing precise thickness determination of the single layers and excluding significant interdiffusion among them. In Figure 3b, a STEM dark-field image of the device section is reported. Layer thicknesses have been determined as described in Methods.

The electric transport properties of the hybrid devices have been studied connecting the Ag and Au stripes and measuring the current flowing as a function of applied voltage by varying the temperature between 135 and 310 K. Considering the rather good energy alignment between reference values for the work functions of Ag and LiF/Au^19,35^ and the estimated HOMO orbital energies for both [Fe(qnal)_2_]_HS_ and [Fe(qnal)_2_]_LS_ (see Figure 3a), the collected data have been analyzed according to SCLC model.^37^ SCLC usually occurs when charges are injected from a metallic electrode to a dielectric such as an insulator or a wide-gap semiconductor: at the interface a space charge region arises which controls the current through the medium. For low voltages and low charge injection, electric current is mainly due to free charges present in the dielectric and follows an ohmic behavior^21^
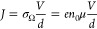
1where *e* is the electric charge, *n*_0_ is the free electron density, μ is the electron mobility, and *d* is the thickness of the dielectric. When the density of injected charges starts becoming comparable to *n*_0_, SCLC injection takes place and, for ideal dielectrics, the current density is described^38^ by *J* ∝ *V*^2^/*d*^3^. Actually, real dielectrics are characterized by the presence of lattice defects, impurities, etc., that can give rise to the presence of electron traps, i.e., states inside the energy gap that can capture electrons by removing them from the conduction band and so reducing the current flow. Taking into account also the presence of electron traps, the relation linking *J* to voltage depends on the energy distribution of the traps.^21^ In particular, we consider the cases of shallow traps and of exponentially distributed traps. In the former, the energy of the traps is close to the edge of the conduction band (*E*_c_ – *E*_t_ ≪ *k*_B_*T*) and *J* is given by
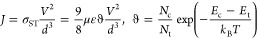
2with ϑ representing the fraction of free electrons obtained as the ratio between the number of states at the bottom of the conduction band, *N*_c_, and the number of trap states, *N*_t_.

If the traps present an exponential energy distribution  the current density is described by the equation^37^
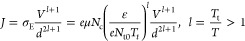
3where the trap distribution temperature *T*_t_ is a parameter related to the energy width of the exponential trap distribution *k*_B_*T*_t_.

**Figure 4:**
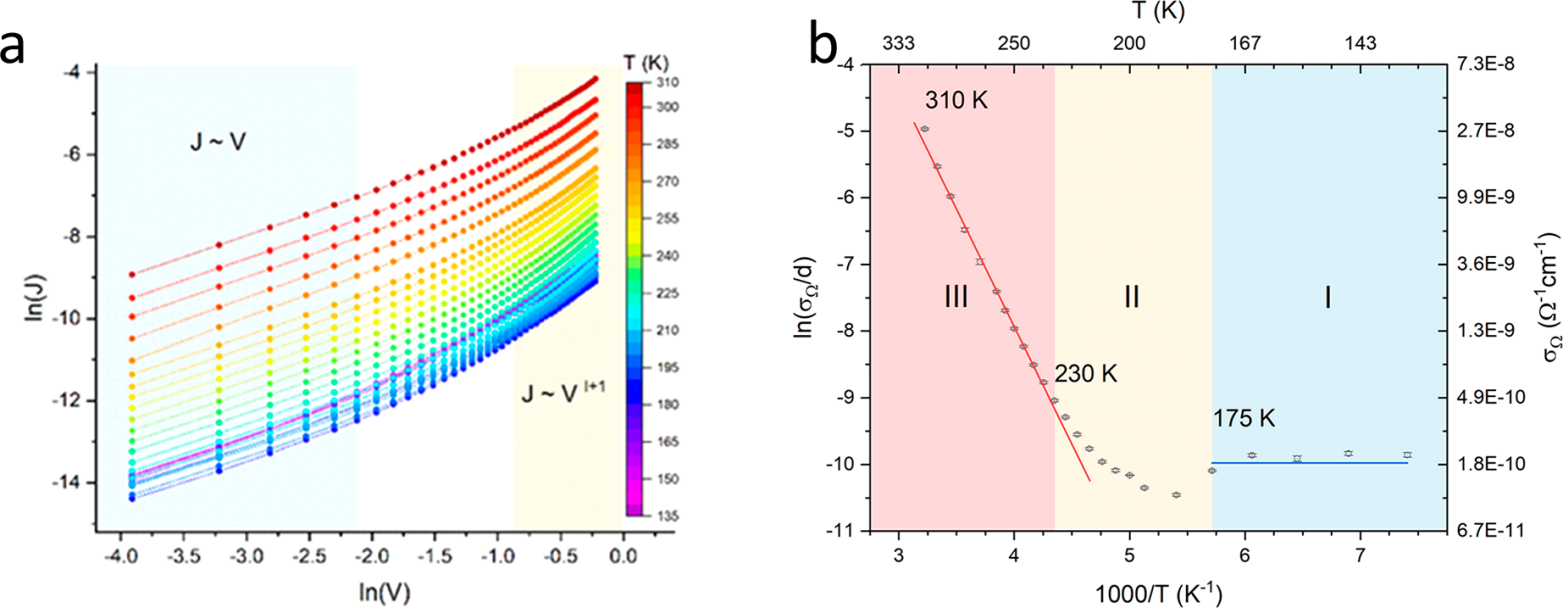
(a) log–log plots of *J*–*V* characteristics at different temperatures (temperature color scaled as per the legend) for one of the realized devices. Low-voltage ohmic and high-voltage SCLC regimes voltage ranges are highlighted. (b) Logarithm of the conductivities measured in the ohmic regime as a function of 1/*T*, revealing three temperature ranges characterized by a different temperature dependence: at low temperatures (*I*), no presence of thermal activation processes are observed, while an Arrhenius activation mechanism is observed at higher temperatures

**Figure 5:**
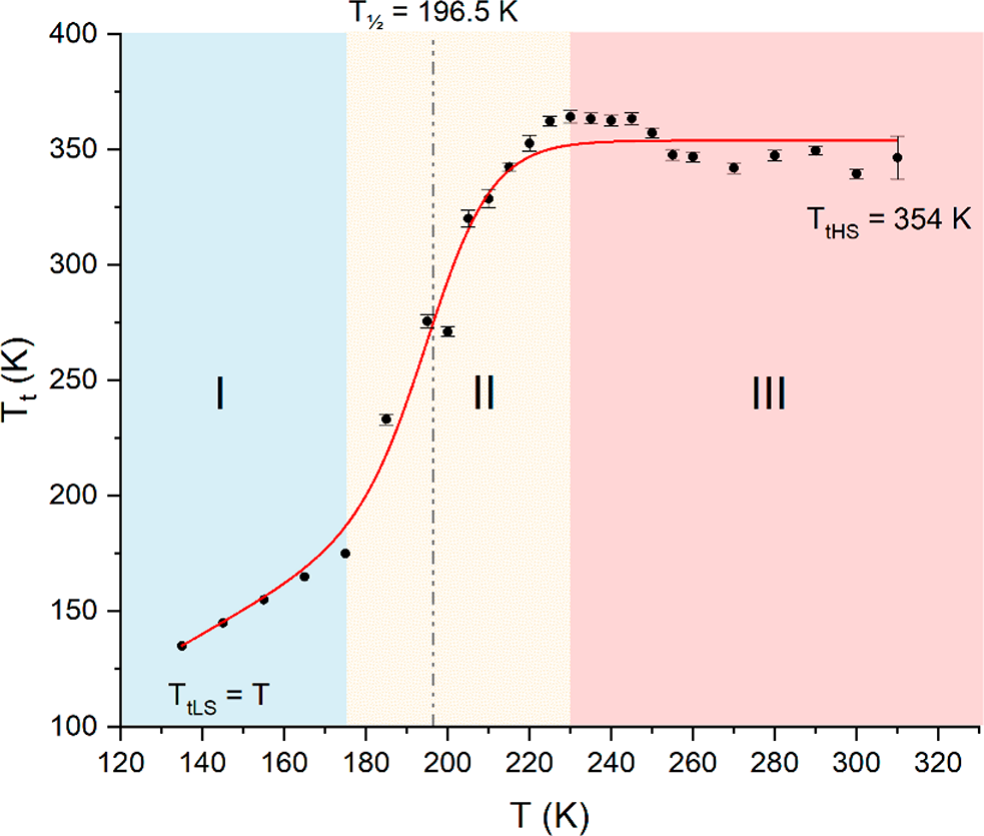
Trend of the *T*_t_ parameter obtained from *J*–*V* analysis (black dots) as a function of temperatures. Transition between shallow traps SCLC (I, *T*_t_ = *T*) and exponential traps distribution SCLC (III) consistent with the LS–HS transition is observed. Result of the fit with a Boltzmann sigmoidal function is shown (red line) together with the related *T*_1/2_.

The *J*–*V* curves collected at different temperatures in the range 135–310 K for one of the devices realized are reported in Figure 4a. All of the *J*–*V* characteristics, as expected in the presence of SCLC, show an ohmic behavior *J* ≈ *V* at low voltages and a superlinear dependence *J* ≈ *V*^α^ (α ≥ 2) at high voltages. Linear fits of ln(*J*)–ln(*V*) curves in the ohmic region, constraining the slope to 1 (see Figure S8), allowed us to obtain from the intercept the values of σ_Ω_ that, once reported as a function of 1/*T* (see Figure 4b), revealed the presence of two distinct regimes characterized by a different dependence of σ_Ω_ on temperature (labeled I and III in Figure 4b) separated by a transition region (II). In the first range (I, *T* ≤ 175 K) no temperature dependence is present, indicating that the measured current is due to free charges present in the material with no contribution of thermally generated free charges. On the contrary, an Arrhenius-type activation mechanism is observed in range III (*T* ≥ 230 K), the fit of which provides an activation barrier of ϕ = 304 ± 3 meV. Referring to eq 1, the temperature trend of σ_Ω_ was ascribed to *n*_0_, , and ϕ was consequently considered as the activation barrier for thermally generated free charges. Furthermore, given the good agreement between the temperature ranges I, II, and III characterizing the σ_Ω_(*T*) behavior with the LS–HS temperature dependence of [Fe(qnal)_2_] (see Figure 1), we link the transport properties observed in intervals I and III to the LS and HS states of [Fe(qnal)_2_], respectively. The results coming from analysis of the currents measured at higher voltages revealed different trends occurring in the temperature intervals I, II, and III. For temperatures lower than 175 K, the current showed a quadratic dependence on voltage (see Figure S9) in accordance with a presence of shallow traps (eq 2), while in the temperature ranges II and III, a more than quadratic dependence is observed, which is consistent with the presence of an exponential trap distribution. In particular, from eq 3 it follows that , so the slope of a linear fit in a log–log plot (see Figure S10) allowed determination of the width of the exponential distribution *k*_B_*T*_t_. The presence of two different energy distributions of traps (shallow and exponential) can be explained as being due to the different packing of the LS and HS states for [Fe(qnal)_2_] and the ensuing different overlap between the aromatic moieties of the ligand in the molecules which are probably crucially associated with the transport mechanism.^23,30^ In Figure 5 are reported the values of the trap distribution temperature *T*_t_ obtained from analysis of the *J*–*V* characteristics in II and III as a function of temperature. For temperature range I, since no trap distribution is involved in the transport mechanism, no temperature trap distribution is defined (see eq 2), and so a fictious value *T*_t_ = *T* was considered imposing to eq 3 a quadratic voltage dependence to resemble that of eq 2. The data confirm a transition, occurring in the temperature range II, from a SCLC mechanism with shallow traps in I (*T*_t_ = *T*) to one with an exponential trap distribution in III (*T*_t_ constant). A Boltzmann sigmoidal function  was used to analyze the data, with *T*_tLS_ = *T* and *T*_tHS_, the values of *T*_t_ in the equilibrium LS and HS states, respectively, while Δ*T* is related to the slope of the transition. Results of the fit led to Δ*T* = 8.1 ± 1.1 K, *T*_1/2_ = 196.5 ± 1.2 K, and *T*_tHS_ = 354 ± 3 K corresponding to a trap distribution width of *k*_B_*T*_tHS_ = 30.4 ± 3 meV. In particular, the *T*_1/2_ value obtained is consistent with the results obtained with SMS spectroscopy, showing a decrease of SCO efficiency once the molecules are deposited as film. Indeed, the *T*_1/2_ value obtained from transport measurements is lower than those evidenced by magnetic and Mössbauer measurements on the bulk. Moreover, comparison with the *T*_1/2_ value obtained with XAS measurements on a film^18^ suggests that the origin of the decrease of SCO efficiency can be addressed to molecular layers closer to the electrode. Indeed, XAS techniques allow characterization of the topmost ∼10 nm, far from the electrode/SCO interface. Moreover, it provides a *T*_1/2_ = 210 K much closer to the bulk value of 225 K as compared to the value *T*_1/2_ = 196.5 K obtained from measurements of transport properties which are determined over the whole [Fe(qnal)_2_] film.

## Conclusions

A hybrid device embedding, in a vertical architecture, an Fe^II^-based molecular thin layer has been developed, and state of the art synchrotron-based Mössbauer characterization of the molecular spin crossover layer and FIB-STEM characterization of the stacked layer have been performed preliminarily to the device functional properties tests. The study of the *J*–*V* characteristics of the device as a function of temperature, corroborated by DFT modeling of the hybrid molecular inorganic junction, revealed the occurrence of a SCLC transport mechanism. Deeper analysis also highlights a transition from a phase characterized by shallow traps in correspondence to the LS state to a phase where an exponential trap distribution determines the transport properties in the temperature range where [Fe(qnal)_2_] is in the HS state. In particular, we obtained evidence that the thin molecular layer of [Fe(qnal)_2_] embedded in the vertical structure presents a fraction of molecules still retaining a SCO behavior and capable of affecting the electronic transport properties of the device. These results confirm the potential of SCO molecules and particularly [Fe(qnal)_2_] as suitable molecules for technological applications where the occurring SCO transition triggers a change in the electronic behavior of the device. We also expect that hybrid devices with sharper and stronger responses can be obtained in the future by increasing the fraction of molecules capable of maintaining SCO behavior once deposited on surfaces, a target which probably requires fine tuning of the structure of the molecules, a high order of the films, and playing on ad hoc developed decoupling layers limiting the detrimental interactions between these molecules and the bottom electrode.

## Methods

### Synthesis

Hqnal and [Fe(qnal)_2_]·CH_2_Cl_2_ were synthesized according to the literature procedures.^22,23^ The ^57^Fe isotopically enriched [^57^Fe(qnal)_2_]·CH_2_Cl_2_ has been prepared according to the procedure used for [Fe(qnal)_2_]·CH_2_Cl_2_ using ^57^FeCl_2_ prepared starting from metallic Fe with an 87% isotopic enrichment in ^57^Fe (see Supporting Information). All other reagents were purchased and used as received. All manipulations were performed under a N_2_ inert atmosphere to avoid oxidation of the Fe(II) salt before complexation. A complete description of the synthetic procedures for both [Fe(qnal)_2_] and [^57^Fe(qnal)_2_] is reported in the Supporting Information (see Figures S1 and S2). PXRD confirmed that the two complexes are perfectly isostructural (Figure S2, Supporting Information)

### Bulk Magnetic Measurements

Magnetization measurements were performed in direct current (dc) scan mode in the 5–300 K temperature range with a scan rate of 2 K/min and with an applied magnetic field of 1.0 T on powdered samples of [Fe(qnal)_2_]·CH_2_Cl_2_ and [^57^Fe(qnal)_2_]·CH_2_Cl_2_ using a Quantum Design Magnetic Properties Measurement System (MPMS) magnetometer equipped with a Superconducting Quantum Interference Device (SQUID) (Figure S2). Magnetization data were corrected for the Teflon sample holder and for the diamagnetic contribution as deduced using Pascal’s constants table.^39^

### Standard Transmission Mössbauer Characterization

Preliminary Mössbauer measurements on a powder sample of [^57^Fe(qnal)_2_] were performed by means of a standard Mössbauer setup in transmission geometry. Mössbauer spectra were collected by means of a Kr–CO_2_ proportional counter, fast electronics for γ-ray spectroscopy, and a Wissel spectrometer, which was run in sinusoidal acceleration mode (*v*_max_ = 2.7 or 4.0 mm/s) and calibrated using a standard metal iron foil. Measurements were carried out between 80 and 290 K using a He-based Oxford flux cryogenic system. The γ-ray source was a 25-mCi ^57^Co in rhodium matrix with Lamb-Mössbauer factor *f* = 0.66, as measured by applying the method described previously.^40^ The intensity of the radiation on the sample, having a focal spot ≈ 1 cm^2^, is on the order of 10^4^ photons/s. The sensitivity of the setup is limited to bulk samples.^41^ Approximately 20.3 mg/cm^2^ of [^57^Fe(qnal)_2_] was used for the measurements.

### Synchrotron-Based Mössbauer Characterization

Nanostructured samples of [^57^Fe(qnal)_2_] on gold were investigated by the Synchrotron Mössbauer Source (SMS) set up at the Nuclear Resonance^42^ beamline ID18 at the European Synchrotron Radiation Facility (ESRF). Collimation and monochromatization is achieved via a multistep optics including the nuclear resonant monochromator obtained with a ^57^FeBO_3_ single crystal.^43,44^ Full width at half-maximum (fwhm) of the ^57^Fe-resonant line was set at a value approximately three times larger than that for a radioactive source (fwhm mean value ≈ 0.34 mm/s), obtaining an intensity of about 1.5 × 10^4^ photons/s. The spot size was ca. 18 μm in both dimensions. The radiation is fully recoilless (it does not contain any background), implying *f*_source_ ≈ 1. The Mössbauer spectra were recorded by collecting the radiation reflected by the surface in a grazing incidence geometry (about 0.1°). This feature, together with the extreme focusing of the beam, provides a 1000-fold amplification factor in the effective thickness (a dimensionless parameter that takes into account the number of Mössbauer active nuclei encountered by the radiation along its path in the sample) with respect to a standard experiment performed at normal or close to normal incidence. Further details can be found in two recent papers in which some of us exploited SMS for characterization of molecular ultrathin layer deposits.^32,45^ Samples for this characterization were a 100 nm film of [^57^Fe(qnal)_2_] grown on a thick polycrystalline gold film deposited on silicon with a 5 nm Ti adhesion layer (SSENS, Enschede, NL) and as a check against the bulk conventional Mössbauer spectra a drop cast film of the compound on the same substrate obtained from a dichloromethane solution. The thickness of this sample was evaluated from the effective thickness obtained from the fit of the SMS spectra and comparing it with that obtained for the 100 nm film (the thickness of the film was determined by AFM). In particular, a ratio of effective thicknesses equal to 3.6 was found, therefore pointing to an average thickness of 320 ± 20 nm for the drop cast film. Spectra were measured at different temperatures in the range 3.0–290 K using the superconducting He-exchange gas cryomagnetic system. For the light-induced excited spin state trapping (LIESST) measurements, the sample holder was modified in order to accommodate a couple of InGaAsP LEDs (Roithner LaserTechnik GmbH, nominal optical power = 2 mW with 545 nm nominal wavelength, decreasing to 531 nm at 10 K).

### Mössbauer Data Analysis

The SMS spectra were interpreted by means of a fitting procedure based on the evaluation of the transmission integral function.^32,45^ At each temperature, the absorption cross-section of the sample was considered as the superposition of two contributions associated with a HS and a LS state, characterized by different values of the hyperfine parameters (isomer shift δ and quadrupole splitting Δ*E*_Q_). Moreover, Gaussian distributions of the LS and HS quadrupole splitting were supposed. A proper fitting of the drop cast and sublimated sample required to include impurity species. Their contribution to the spectra is on the order of 10% and 20% for the drop cast and 100 nm thick samples respectively. The same fitting procedure was used to interpret the spectra of the bulk sample measured with standard Mössbauer spectroscopy, except for replacing in the transmission integral function the line shape of the source and the sample effective thickness with those of the standard Mössbauer setup.^32^ The core of the fitting procedure is represented by the LMDIF routine of the MinPack library (www.netlib.org). Moreover, the standard deviations on the fit parameters are evaluated from the diagonal terms of the variance–covariance matrix, which is a fit procedure output.

### Electrochemical Analysis

The electrochemical experiments were carried out using an Autolab PGSTAT101 potentiostat. The experiments were performed under an inert atmosphere of argon using a three-electrode configuration in a single-compartment cell. The electrodes consisted of a platinum disc as the working electrode, a silver wire coated with silver chloride as the reference electrode, and a platinum wire as the counter electrode. A 0.1 M solution of *n*Bu_4_PF_6_ (Fluka, electrochemical grade) in dichloromethane (Honeywell Riedel-de Haën, *purissimum*) was used as supporting electrolyte. Standard cyclic voltammetry experiments (Figure S5) and square wave voltammetry experiments (Figure S5) were performed at 25, 50, 100, and 200 mV s^–1^ and were done both with and without ferrocene as an internal reference. The potentials are quoted versus the Fc/Fc^+^ redox couple. For estimation of the ionization potentials, we used the literature value *E*_1/2_(Fc/Fc^+^) = 0.640 V vs NHE.

### Theoretical Modeling

The orbital energies were estimated from density functional theory calculations with the Gaussian09^46^ program using the restricted and unrestricted B3LYP functionals^47−50^ for the low-spin and high-spin complexes, respectively. The geometry of the complexes was first preoptimized with the LanL2DZ basis set^51−54^ followed by geometry optimization with the 6-31G(d,p) basis set,^55−58^ and finally, single-point energy calculations were performed with the 6-31++G(d,p) basis set.^59^

### Device Preparation

Ultraflat Ag metal stripes were prepared using a combination of standard lift-off and template-stripping processes. A 300 nm thick layer of LOR-3B lift-off resin (Microchem) was spin coated on a high-quality Si (100) wafer (WAS4P1020, Neyco) at 3000 rpm for 45 s, and the wafer was subsequently baked at 180 °C for 15 min. A 2 μm thick layer of positive photoresist (S1818, Shipley) was then spin coated on top of the LOR3B at 4000 rpm for 30 s, and the wafer was then baked at 115 °C for 60 s. The resist was then exposed through a high-resolution soft photomask (Selba) with a UV-Kub 2 (Kloe) LED exposure tool (8 s at 100% power) and developed in MF-319 (Microposit) for 40 s under gentle agitation. The developed wafer was then rinsed carefully with deionized water and dried under a stream of nitrogen. After drying, it was descummed with air plasma for 5 min (Harrick Plasma). Two hundred nanometers of silver was then evaporated on top of the patterned wafers with a thermal evaporator (Plassys) at a rate of 1–2 Å/s, the lift off was performed in acetone with gentle agitation until complete removal of the unwanted metallic film, and the excess LOR-3B resist was removed by gentle agitation in MF-319. To allow for an easy peeling of the stripes, we modified the SiO_*x*_, selectively, by vapor silanization with a fluorosilane (1*H*,1*H*,2*H*,2*H*-perfluorooctyl-trichlorosilane, Sigma-Aldrich) (see the processing scheme in Figure S6 in the Supporting Information). We cast a UV-curable optical adhesive (OA, NOA 61, Norland) to glue the stripes to a glass chip, and after UV exposure we cleaved the Ag/adhesive/glass composite from the wafer to obtain the array of electrodes. The molecular sublimations were performed in a homemade effusion cell. A crucible was filled with the powder of [Fe(qnal)_2_]·CH_2_Cl_2_, and once the pressure reached the 10^–7^ mbar range, the temperature was gently raised up to the sublimation temperature. The deposition rate was monitored by a quartz microbalance (QCM) and stabilized in the 0.4 nm/h range at a temperature of 570 K. The QCM calibration was determined by deposition of a 50 nm thick film of [Fe(qnal)_2_] on an ultraflat silicon wafer through a patterned TEM grid and by evaluating the thickness of the unmasked area of that film ex situ (Figure S6) by an NTMDT P47-pro AFM setup equipped with NSC36 micromash tips. The vertical device was finalized (Figure S7) by evaporating 40 nm of [Fe(qnal)_2_] on top of 50 μm wide template-stripped Ag wires (obtained as described above) and subsequent evaporation of 10 nm of LiF, deposited using an e-beam heated cell, above which a gold crossbar (width ca. 250 μm and height ca. 35 nm) was realized with evaporation through a metallic mask.

### FIB-STEM Analysis

TEM lamella preparation and STEM characterization methodologies were used to acquire a stratigraphic profile of the device, thus enabling one to check its architecture (thickness and roughness of the layers). Lamella preparation and STEM analysis were performed in a single workflow using a TESCAN GAIA 3 FIB/SEM. The microscope, located at the Electron Microscopy Facility (CE.M.E.) of the CNR in Florence, is equipped with a Triglav electron column and a Cobra Ga-ion column. An 8 × 4 × 1 μm^3^ lamella (length × depth × thickness) was extracted from the surface of one of the complete devices; the lamella was then thinned down to less than 40 nm prior to characterization by the built-in STEM detector. STEM images were analyzed with the software Gwyddion,^60^ determining the thicknesses of the layers measured, within the experimental error and the distances between contiguous inflection points in the vertical intensity profiles.

### Transport Measurements

Electric transport measurements on devices were performed employing a homemade cabled sample holder in order to place the sample inside a PPMS (Quantum Design), which allowed control of the sample temperature. Gold wires and conductive silver paint were used for electric connections of the samples. *I*–*V* measurements were performed at different temperatures in the range 135–310 K, making use of a source-meter unit (Keithley SMU 2601) to supply voltages (in the range 0–1 V), while the currents were measured with an electrometer (Keithley 6514) in a 2-wire configuration.

## Supporting Information Available

Additional characterization data: synthesis, bulk magnetic, and powder XRD spectra, AFM images, device preparation, Mössbauer characterization, voltammetric data, and electric transport characterization (PDF)
