# Supplementary material for: Space Charge-Limited Current Transport Mechanism in Crossbar Junction Embedding Molecular Spin Crossovers
Source: ACS Appl Mater Interfaces. 2020 Jun 18;12(28):31696–705. doi: 10.1021/acsami.0c07445 (PMC8008390; doi:10.1021/acsami.0c07445)
Supplement: Supplementary file 1 — am0c07445_si_001.pdf [file am0c07445_si_001.pdf]

Supporting Information

Giuseppe Cucinotta,[1,‡,] Lorenzo Poggini,[1,2,‡,*] Niccolò Giaconi,[1] Alberto Cini,[3] Mathieu Gonidec,[2] Matteo Atzori,[1] Enrico Berretti,[4] Alessandro Lavacchi,[4] Maria Fittipaldi,[3] Aleksandr I. Chumakov,[5] Rudolf Rüffer,[5] Patrick Rosa,[2] Matteo Mannini.[1]*

1) Department of Chemistry "U. Schiff" and INSTM Research Unit, University of Florence, Via della Lastruccia 3-13, 50019 Sesto Fiorentino (FI), Italy

2) CNRS, University of Bordeaux, ICMCB, UMR 5026, 33600 Pessac, France

3) Department of Physics and Astronomy and INSTM Research Unit, University of Florence, Via Sansone 1, 50019 Sesto Fiorentino (FI), Italy

4) Institute for Chemistry of OrganoMetallic Compounds (ICCOM-CNR), Via Madonna del Piano, 50019 Sesto Fiorentino (FI), Italy

5) ESRF-The European Synchrotron, Avenue des Martyrs 71, 38000 Grenoble, France

Synthesis

Synthesis of N-(8'-quinolyl)-2-hydroxy-1-naphthaldimine (Hqnal). A solution of 8-aminoquinoline in EtOH (2 g, 13 mmol in 30 mL) was added to a solution of 2-hydroxy-1-naphthaldehyde in EtOH (2.24 g, 13 mmol in 40 mL) under slight heating. The resulting orange mixture was stirred for 15 minutes. After cooling to room temperature, the precipitate was filtered and washed with EtOH and diethyl ether. The product was dried under reduced pressure to obtain a microcrystalline orange powder (2.41 g, yield 56%). CHN, Found (Calcd.): C 79.31 (80.52); H 4.37 (4.73); N 9.13 (9.39). FT-IR: (cm^{-1}, KBr): 3057 vw, 3037 vw, 1624vs, 1591m, 1567w, 1538s, 1488m, 1471m, 1395w, 1355m, 1303s, 1268w, 1208w, 1135vw, 1081vw, 1037vw, 959vw, 856vw, 820w, 788w, 746w, 703vw, 596vw, 554vw, 481vw, 403vw. ^{1}H-NMR (400 MHz, CD_2Cl_2): 9.1 (dd, 1H, J = 4.2 Hz, J = 1.6 Hz), 8.2 (dd, 1H, J = 8.3 Hz, J = 1.6 Hz), 8.0 (d, 1H, J = 8.3 Hz), 7.7 (d, 1H, J = J = 9.4 Hz), 7.3 (t, 1H, J = 7.9 Hz), 6.9 (d, 1H, J = 9.5 Hz).

Synthesis of ^{57}Fe(II) chloride. 16 mL of a degassed solution of HCl in MeOH (5.5 M) was added to an isotopically enriched ^{57}Fe plate (50 mg, 0.90 mmol) under a constant flow of N_2 at 60° C and the mixture stirred for 1 hour. After cooling at room temperature, an additional equivalent quantity of acidic solution was added, and the mixture allowed to react for additional 48 hours. The resulting slight yellow solution, which is indicative of the presence of $^{57}Fe^{3+}$, was concentrated by heating at 100° C. The $^{57}Fe^{2+/3+}$ iron salt was dissolved in MeOH and placed again to starting conditions (60° C, N_2 atmosphere). An additional amount of ^{57}Fe (35 mg, 0.63 mmol) and few drops of degassed acidic solution were added to initiate the reduction of $^{57}Fe^{3+}$ to $^{57}Fe^{2+}$. After 3 hours the solution became colourless, indicating that only $^{57}Fe^{2+}$ chloride was present in solution, thus, the

reaction was stopped by concentrating the mixture at 160° C under vacuum to obtain a sand color powder of $^{57}FeCl_2$ (220 mg, 86%).

Synthesis of [^{57}Fe(qnal)$_2$]·CH_2Cl_2. A mixture of Hqnal (150 mg, 0.5 mmol) and Et_3N (50 mg, 0,5 mmol) was dissolved in 30 mL of CH_2Cl_2. A solution of ^{57}Fe(II) chloride (31.6 mg, 0.25 mmol) in 20 mL of degassed MeOH was added dropwise to the first mixture under nitrogen atmosphere and stirred for 30 minutes at room temperature. The brown precipitate was filtered, then washed with CH_2Cl_2 and diethyl ether to obtain a brown microcrystalline powder (95 mg, 51%).

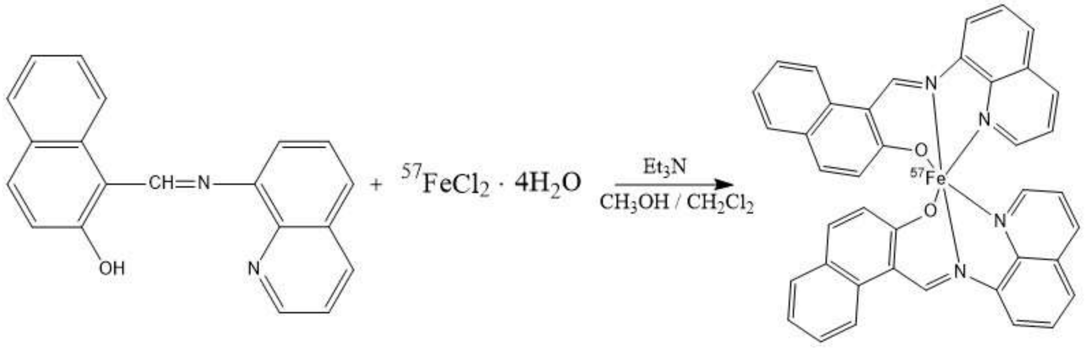

Figure S1. Scheme of the synthesis of [^{57}Fe(qnal)$_2$]·CH_2Cl_2

Powder X-ray diffraction and Bulk magnetic characterization

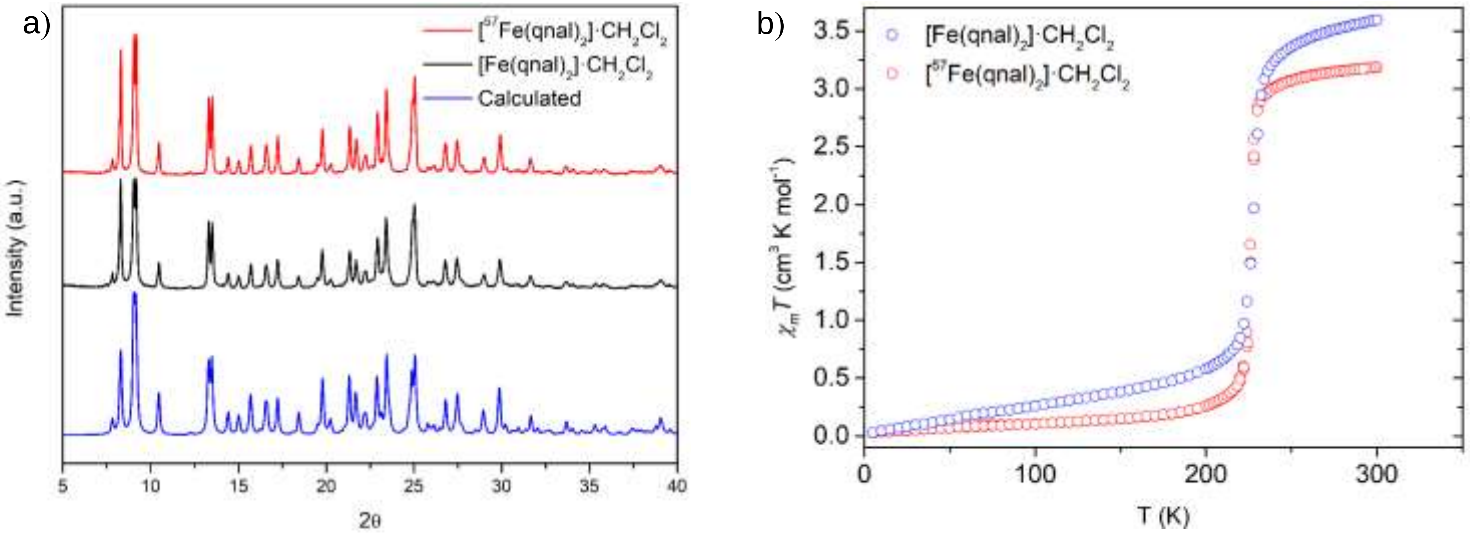

Figure S2. a) Powder XRD spectra of [^{57}Fe(qnal)$_2$]·CH_2Cl_2 (red) and of [Fe(qnal)$_2$]·CH_2Cl_2 (black). The experimental data are in accordance with the calculated spectra (blue). b) Magnetic susceptibility measurements of [Fe(qnal)$_2$]·CH_2Cl_2 and of the enriched compound [^{57}Fe(qnal)$_2$]·CH_2Cl_2.

Mössbauer characterization

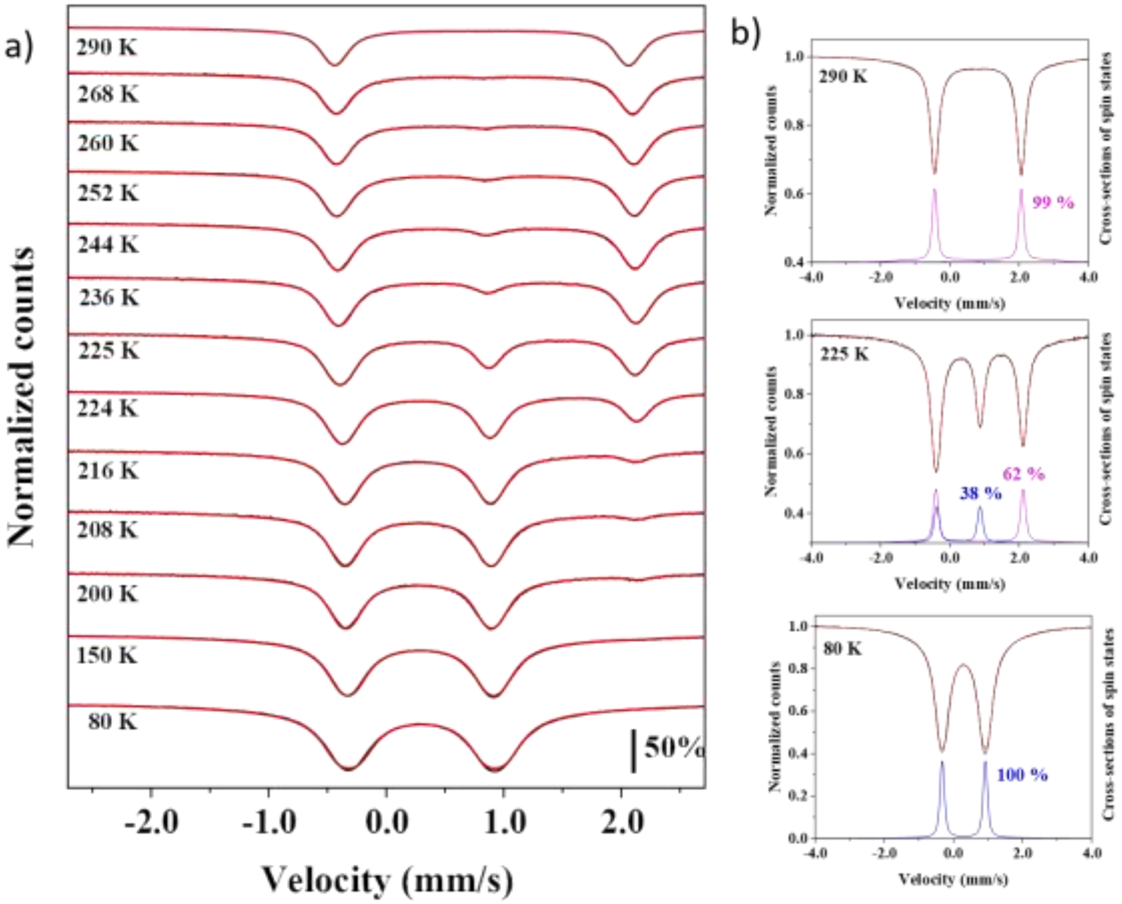

Figure S3. a) transmission Mössbauer spectra (black lines) of the powder sample of [$^{57}Fe(qnal)_2$] as a function of temperature and corresponding fit (red lines). b) transmission Mössbauer spectrum (black line) and corresponding fit (red line) and HS (magenta line) and LS (blue line) contributions at selected temperatures.

Table S1. *[57]Fe Mössbauer parameters of the HS and LS states extracted from the fit of the spectra of the samples at the highest and lowest temperature: isomer shift with respect to α-Fe (**δ**), quadrupole splitting (**ΔE_Q**) and Gaussian distribution of quadrupole splitting (**σ**). The calculated HS fraction (%HS) is also shown. The estimated error in the reported parameters comes from the fit procedure. The interpretation model is such that the inhomogeneities are represented by σ and as a result the parameters **δ** and **ΔE_Q** are quite well determined.*

Sample (method)	***T*** (K)	LS state **δ** (mm/s)	LS state **ΔE_Q** (mm/s)	LS state **σ** (mm/s)	HS state **δ** (mm/s)	HS state **ΔE_Q** (mm/s)	HS state **σ** (mm/s)	%HS
Bulk (standard Mössbauer)	290	0.335	0.98	0.03(2)	0.81207(9	2.5009(2)	0.0450(2)<br>0.941(4)[a]	99.5(4)
	80	0.3026(1)	1.2477(3)	0.0381(9)<br>0.950(8)[a]				0
Dropcast (SMS)	290	0.40(7)	0.9(1)	0	0.890(3)	2.496(6)	0.065(5)	97(6)
	3.0	0.381(1)	1.198(1)	0				0
100 nm (SMS)	284	0.15(3)	1.1	0	0.945(8)	2.33(2)	0.136(9)	88(7)
	3.0	0.32(1)	1.24(2)	0	0.993(8)	2.42(2)	0.107(8)	63(4)

[a] Two sites were considered having same **δ** and **ΔE_Q** and different **σ**: the site with the largest **σ** contributes to the spectrum by about 30 % at high *T* and by 9 % at low *T*.

Parameters with no error were kept fixed in the fitting procedure, as a direct estimation of them was not possible, given their minor contribution to the spectra. Moreover, the evaluation of the LS state parameters of the 100 nm sample at 284 K is hampered by the decreased quality of the SMS spectrum although a decreasing trend of δ upon increasing the temperature is evidenced by spectra acquired al lower temperatures.

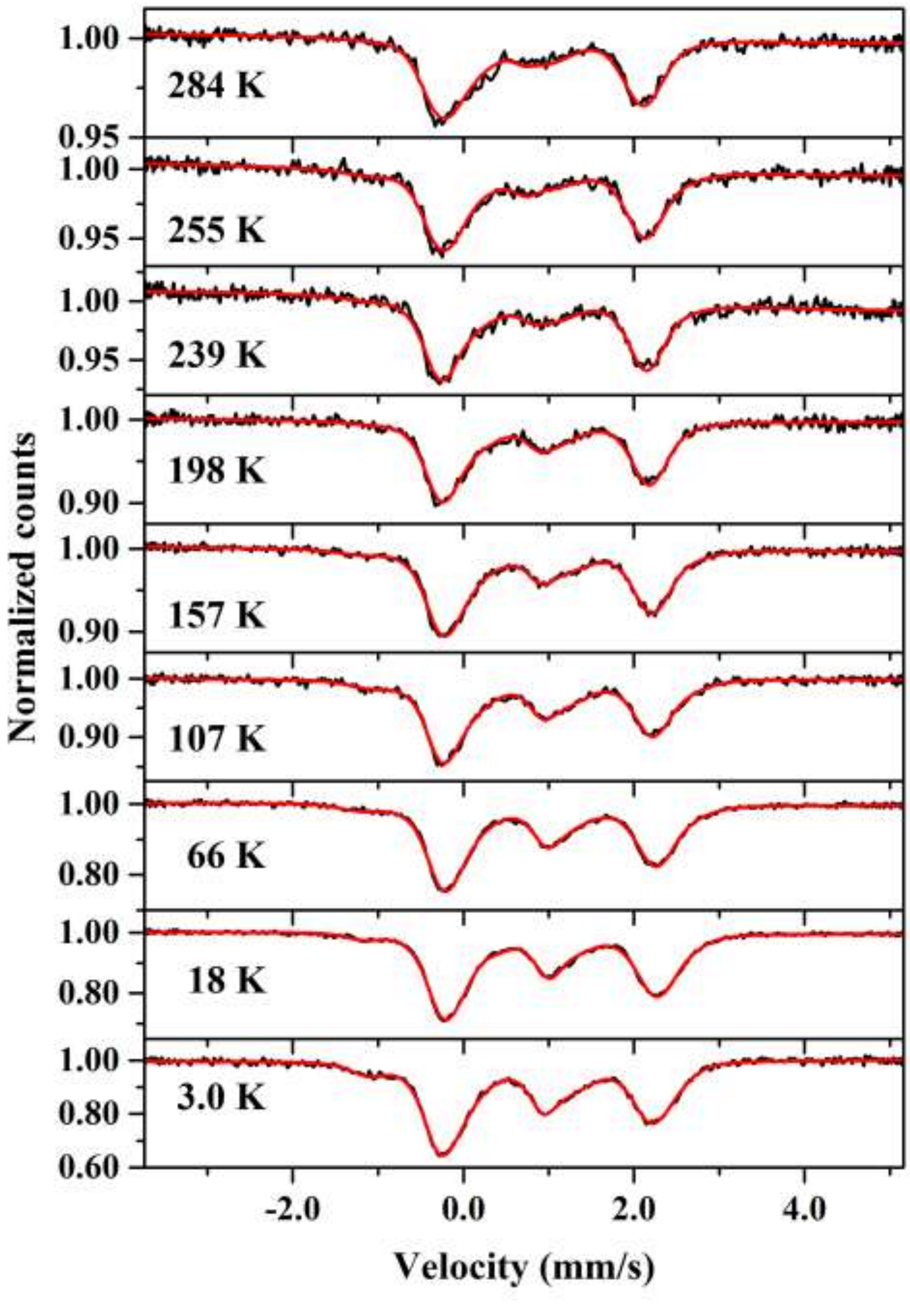

Figure S4. SMS spectra (black lines) of the 100 nm sublimated sample of $[^{57}Fe(qnal)_2]$ as a function of temperature and corresponding fit (red lines).

Electrochemistry

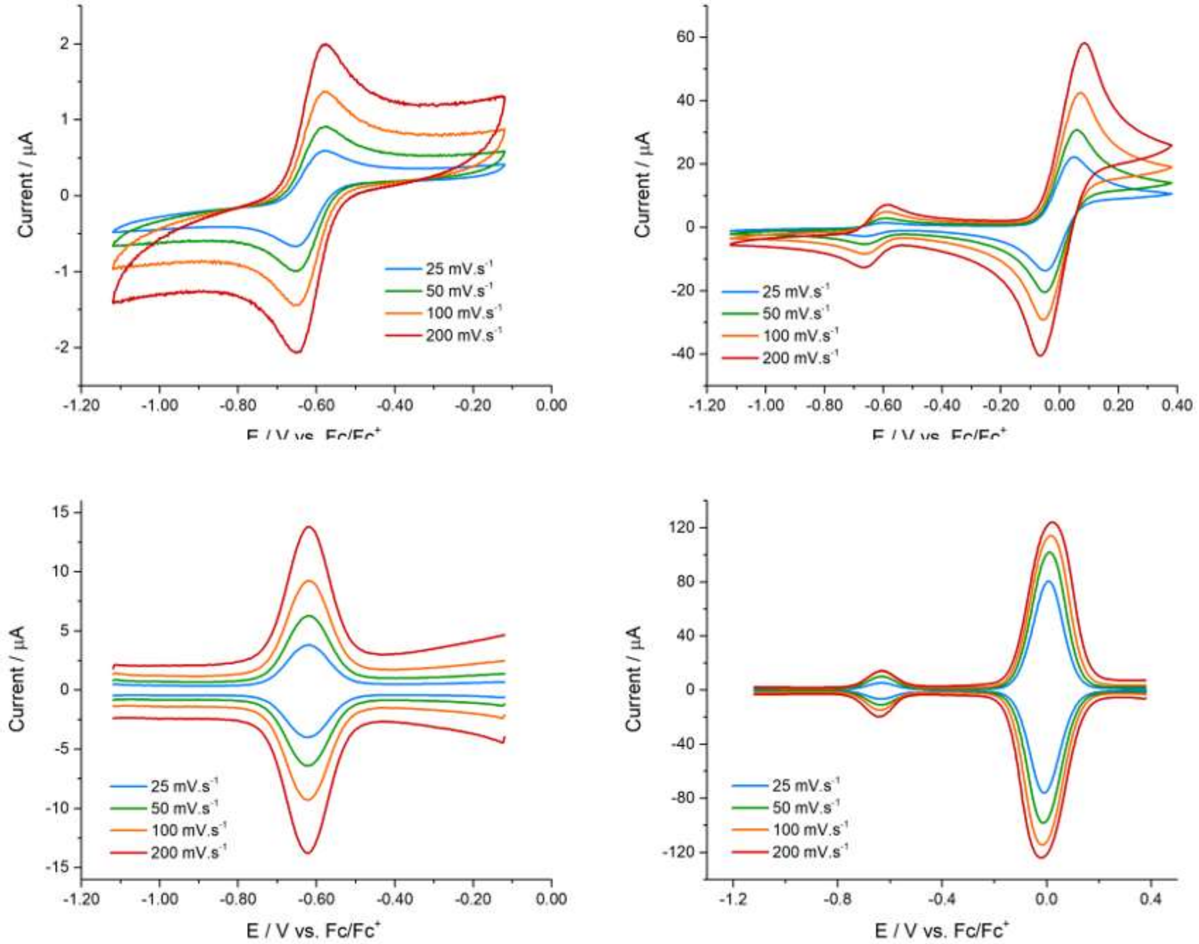

Figure S5. Room-temperature cyclic (top) and square wave (bottom) voltammetry of [Fe(qnal)$_2$] in dichloromethane with 0.1M NBu_4PF_6 as supporting electrolyte at various scan rates, performed without (left) and with some ferrocene (right) as an internal reference, using a Pt disc working electrode, an Ag wire coated with AgCl as a pseudo reference electrode, and a Pt wire as counter electrode.

AFM characterization

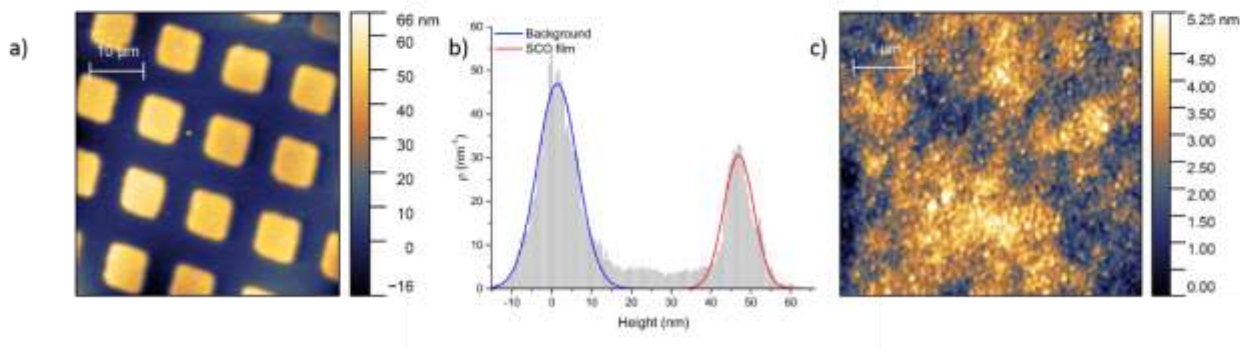

Figure S6. a) AFM topography image of a patterned film of [Fe(qnal)$_2$] sublimated on a silicon wafer through a TEM grid with a target thickness of 50 nm; b) Height distribution histogram from the AFM topography image showing an actual film thickness of ca. 47 ± 2.5 nm. The blue and red lines are Gaussian fits to the height distribution of the background and SCO film, respectively; c) 5×5 µm AFM topography image of [Fe(qnal)$_2$] on Au (111) surface.

Device preparation

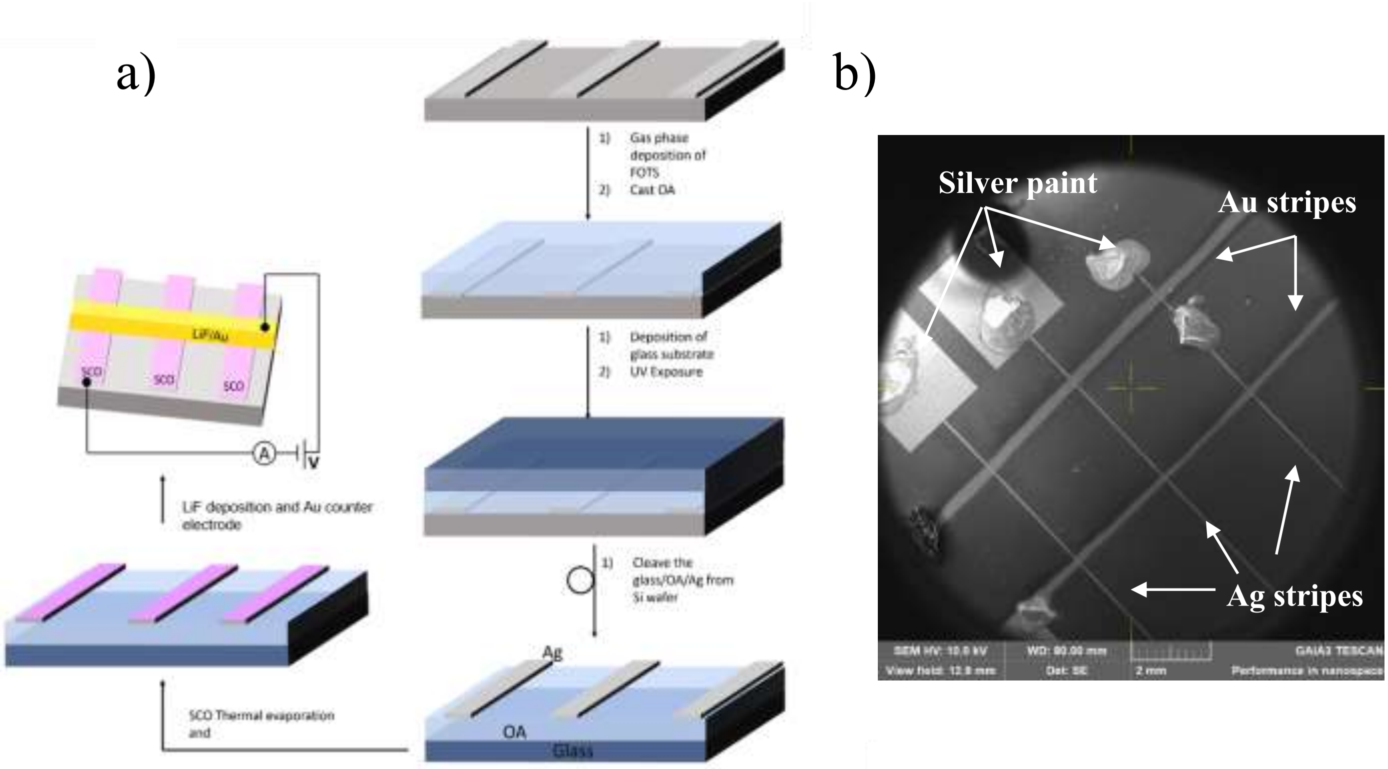

Figure S7. a) Fabrication of SCO-based vertical junctions for temperature-dependent measurements. We patterned template-stripped silver stripes as the bottom electrodes by thermal evaporation of silver on an ultra-flat silicon wafer (as described in the methods section). The SiO_x surface has been modified, by vapour silanization with a fluorosilane. We cast a UV-curable optical adhesive (OA) to glue the Ag stripes to a glass chip, and after UV exposure; we cleaved the Ag/adhesive/glass composite from the wafer to obtain the array of electrodes. After thermal evaporation of $[Fe(qnal)_2]$ over the substrate, we finished the vertical junction by LiF and Au evaporation. b) SEM picture of a real device containing 6 vertical junctions obtained from three silver stripes crossed by two gold stripes. Gold wires for electrical measurements were connected using silver paint.

Electric transport characterization

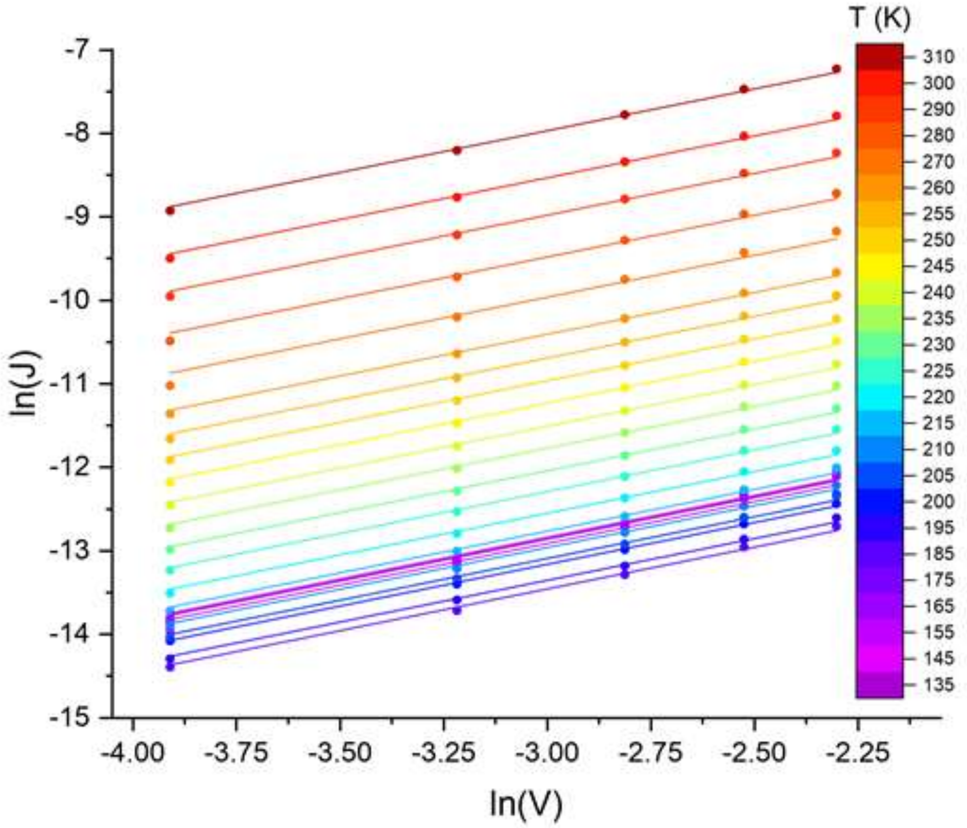

Figure S8. Log-log plot of J-V characteristics at different temperatures in the ohmic regime at low voltages (dots) and results of linear fits (sold lines).

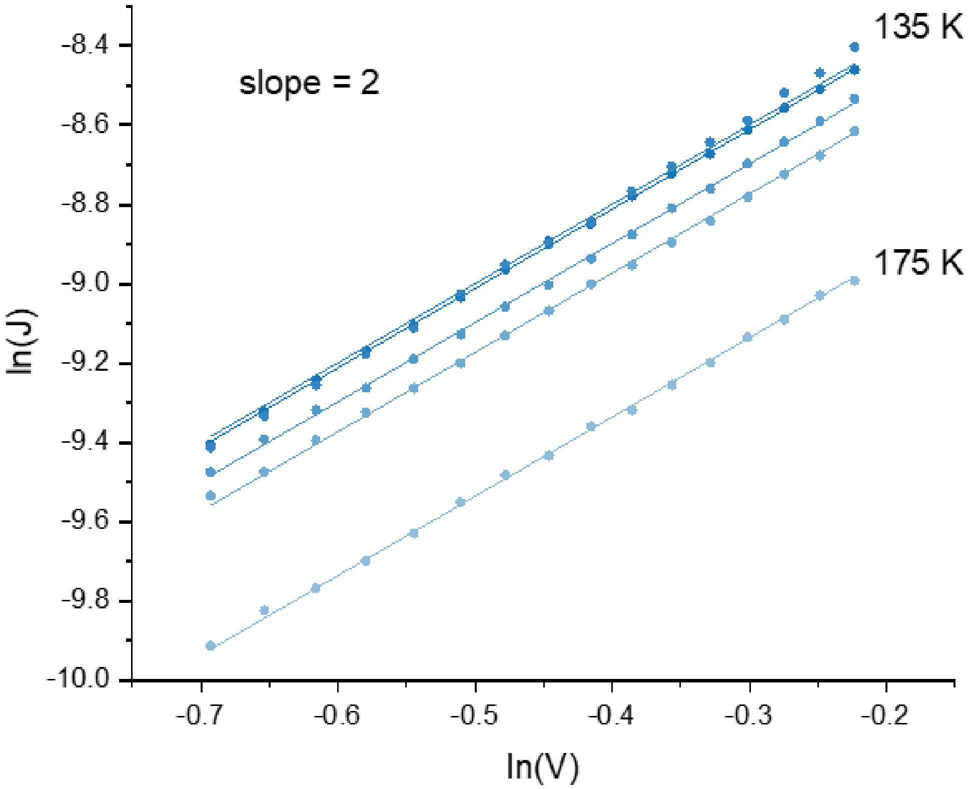

Figure S9. Log-log plots of J-V characteristics in the SCLC regime measured in temperature range I (dots) and results of fits to a quadratic voltage dependence (solid lines).

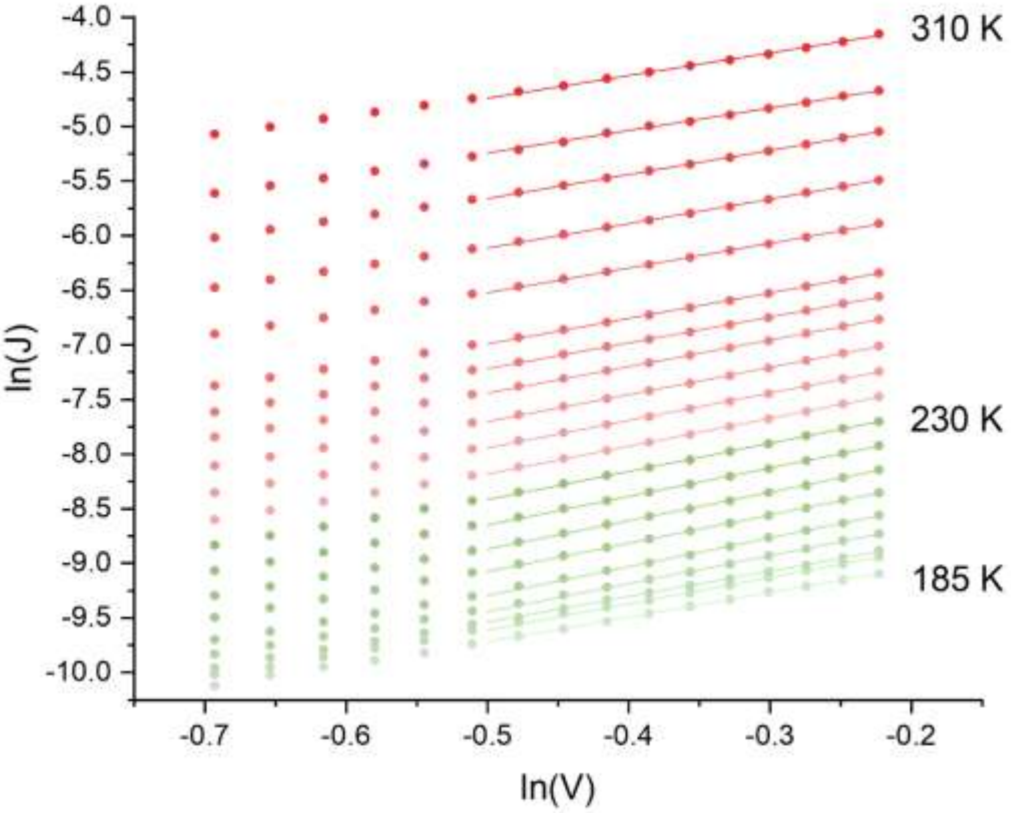

Figure S10. Log-log plot of *J*-V characteristics in the SCLC regime measured in the temperature ranges II and III (green and red dots respectively) and results of linear fits (solid green and red lines respectively).
